# Antibiograms from community-acquired uropathogens in Gulu, northern Uganda - a cross-sectional study

**DOI:** 10.1186/1471-2334-13-193

**Published:** 2013-04-29

**Authors:** Charles O Odongo, Denis A Anywar, Kenneth Luryamamoi, Pancras Odongo

**Affiliations:** 1 Department of Pharmacology & Therapeutics, Faculty of Medicine, Gulu University, P.O. box 166, Gulu, Uganda; 2 Department of Biochemistry and Molecular Biology, Faculty of Medicine, Gulu University, Gulu, Uganda; 3 Department of Medical Microbiology & Immunology, Faculty of Medicine, Gulu University, Gulu, Uganda; 4 Department of Internal Medicine, Faculty of Medicine, Gulu University, Gulu, Uganda

**Keywords:** Antibiograms, Antibiotic resistance, Uropathogens, Empirical treatment, Uganda

## Abstract

**Background:**

Urinary tract infections (UTI) are common in clinical practice and empirical treatment is largely employed due to predictability of pathogens. However, variations in antibiotic sensitivity patterns do occur, and documentation is needed to inform local empirical therapy. The current edition of the Uganda Clinical Guidelines recommends amoxicillin or cotrimoxazole as choice drugs for empirical treatment of community-acquired UTI. From our clinical observations, we suspected that this recommendation was not effective in our setting. In order to examine validity, we sought to identify bacteria from community-acquired infections and determine their susceptibility against these antibiotics plus a range of potentially useful alternatives for treatment of UTI.

**Methods:**

A cross-sectional study of mid-stream urine collected from 339 symptomatic patients over a three-month period at Gulu regional referral hospital. Qualitative culture and identification of bacteria and antibiotic sensitivity testing using the modified Kirby-Bauer disk diffusion method was done. Participants’ demographic and clinical characteristics were collected using a standard form. Results were analyzed by simple proportions among related variables and confidence intervals computed using binomial exact distribution.

**Results:**

Eighty two cultures were positive for UTI. *Staphylococcus spp* (46.3%) and *Escherichia coli* (39%) were the most common pathogens. There was high resistance to cotrimoxazole (73.2%), nalidixic acid (52.4%) and amoxicillin (51.2%). The most favorable antibiograms were obtained with gentamicin, amoxicillin-clavulanate and levofloxacin where 85.4%, 72.0%, 67.1% of isolates respectively, were either sensitive or intermediate. Only 51% of isolates were sensitive to ciprofloxacin.

**Conclusion:**

There was high resistance to most antibiotics tested in this study. The recommendations contained in the current edition of the Uganda Clinical Guidelines are not in tandem with antibiotic sensitivity pattern of uropathogens seen in our setting. Amoxicillin-clavulanate or gentamicin should be considered for replacement of amoxicillin and cotrimoxazole for empirical treatment of UTI in our setting.

## Background

Urinary tract infections (UTI) are a common medical problem encountered in medical practice. Females are more prone to infection than males, a phenomenon explained by their short urethra and its anatomical proximity to the anal orifice [1,2]. Because uropathogens largely originate from colonic flora [3], they are easy to predict. This is the rationale for empirical treatment in community-acquired urinary tract infections (CA-UTI) [1,4]. However, empirical treatment limits opportunities for surveillance of antibiotic resistance among pathogens that cause community-acquired UTI. If regularly updated to match changing susceptibility patterns, empirical treatment is also a convenient strategy for effective resource utilization. This is especially true in developing countries where resource constraints mean that it is often impractical to routinely perform antibiotic sensitivity tests [5]. Reports of uropathogens resistant to previously effective antibiotics have emerged globally in recent years [6-18]. The situation is especially dire in Africa where irrational antibiotic practices are common [18]. Variations in antibiotic resistance patterns are known to occur across different geographical regions, even within the same country [16,19]. Such variations must be well documented so as to inform local empirical treatment as well as foster rational antibiotic use.

The current edition of the national treatment guidelines in Uganda recommends the use of amoxicillin or cotrimoxazole for empirical treatment of CA-UTI [20]. However, based on our clinical experience at Gulu regional hospital, we suspected that resistance to these antibiotics was possibly high. Therefore, continued empirical use was likely to increase chances of treatment failure, leading to unnecessary patient suffering and increased healthcare costs in the long run. The broad aim of this study was to examine the validity of the current recommendations for empirical treatment of CA-UTI as per the national guidelines. Specifically, we set out to identity uropathogens from community-acquired infections at Gulu regional hospital and thereafter, determine their resistance profiles against the currently recommended antibiotics. In addition, we sought to determine resistance profile against a range of potentially useful alternatives for treatment of CA-UTI in our setting.

## Methods

### Study design

This was a cross-sectional study employing both qualitative and quantitative methods. All outpatients (≥18 yrs) with symptoms suggestive of a UTI were consecutively recruited into the study. Symptoms of interest included supra-pubic pain, a burning sensation while voiding (dysuria), urine frequency and urge incontinence. A pre-designed standard form was used to record relevant socio-demographic and medical history information, as well as important clinical and laboratory findings. Participants were asked to provide an on-the-spot urine sample for investigation to determine true cases of UTI. Bacterial isolates from true infections were identified and subjected to antibiotic susceptibility testing using the Kirby-Bauer disk diffusion method [21]. The recommended antibiotics, plus a range of potentially useful alternatives were included in the tests which were performed at the microbiology laboratory of the Faculty of Medicine, Gulu University.

### Urine sample collection

Mid-stream (clean-catch) urine samples were collected by participants under the supervision of nurses and midwives serving as research assistants. These were trained on how to instruct and guide urine collection as per standard methods [22]. Mindful of the cultural sensitivity of the procedure, we employed research assistants who were conversant with the local Luo dialect and cultural experience. Participants were led into a clean toilet facility and asked to wash their hands with soap and water prior to urine collection. They were handed a well-labeled, sterile, wide mouth urine container (50 ml) and accordingly instructed. Labeling consisted of participant study number, sex, and date of urine collection. Briefly on the collection method, the uncircumcised male was told to retract the foreskin on the glans penis prior to collection. He was asked to void the first bit of urine into the toilet pan before collecting the mid-portion (starting approximately 2 s from beginning of flow) and then finally voiding the last bit again into the toilet pan. He then closed the bottle and passed it on to the research assistant for storage. In the case of females, the research assistant gently separated the labia minora and wiped the area around the urethral orifice (from front to back) using sterile gauze dipped in normal saline. This procedure was repeated with the labia held apart before collection of urine as described above. All samples were immediately stored in an ice box and transported to the laboratory within three hours of collection.

### Determination of true infection and identification of uropathogens

Urine samples were registered in the record book and given serial laboratory numbers. The consideration of a sample as ‘true UTI’ was arrived at in a two-stage screening process involving microscopic enumeration of urine leucocytes followed by quantitative urine culture. This approach was adopted because neither approach alone was considered sufficient to conclusively label a case as ‘true UTI’. For instance, urinary schistosomiasis or prior antibiotic use, were likely to confound findings from the above methods respectively. First, one microliter (1 μL) of a well mixed urine sample was aspirated into an improved Neubauer counting chamber (Marienfeld-GmbH, Germany) and examined with a light microscope under X 400 magnification. A finding of at least 5 and 10 leucocytes per microliter in males and females respectively was considered UTI basing on previous recommendations [23]. All samples that were positive by this criterion were further subjected to urine culture in the second stage. A 10 μL plastic loop was used to pick a well mixed urine sample which was plated for quantitative culture on Nutrient Agar (BioMerieux/Hi Media) and CLED Agar (BD Diagnostic Systems, Germany) prepared according to standard methods [24]. The use of two media was aimed at isolating all possible bacteria as well as using their unique colony growth characteristics to aid identification. All plates were inverted and incubated at 35°C (± 1) for 18 to 24 hours with each resultant colony considered to represent 100 CFU/ml. A cutoff point of 10^5^ CFU/ml was considered significant growth as long as the culture was pure [24]. All urine samples that had significant leucocyturia and thereafter significant bacterial growth were considered true UTI. In addition, samples that had significant leucocyturia but less than 10^5^ CFU/mL were considered true UTI if there was a positive history of antibiotic use prior to presenting at the hospital. Unique colony growth characteristics, gram staining and a battery of biochemical tests were used to identify uropathogens. These included sugar fermentation using TSI agar, litmus milk decolorization, citrate utilization, indole production, the methyl red test, as well as catalase, coagulase and urease tests. All tests were performed according to standard methods described elsewhere [24].

### Preparation of Mueller-Hinton agar and antibiotic susceptibility assays

Antibiotic susceptibility was done on Mueller-Hinton II agar (Oxoid, UK) according to the Kirby-Bauer method modified in accordance with current guidelines from the Clinical & Laboratory Standards Institute (CLSI) [25]. Preparation of Mueller-Hinton II agar was by dissolving 38 g/L as per manufacturer’s recommendations. All agar preparations were fully dissolved and autoclaved at 121°C for 15 minutes. After sterilization, 25 ml was gently poured onto each sterile petri dish (90 mm diameter) placed on a horizontal work top and allowed to cool to room temperature. This corresponds to an agar depth of 4 mm [24]. The plates were then inverted and stored in sealed plastic bags at 4–8°C until use, in any case not longer than 15 days. Quality control of each batch was ensured by testing Enterococcus faecalis (ATCC 2922) against cotrimoxazole. Prior to inoculation and placement of antibiotic disks, sterility of each plate was ensured by overnight incubation (35 ± 1°C) in which no growth occurred.

Three to five morphologically similar colonies of each organism (aged 18–24 h) were touched with a sterile loop and inoculated in 4 ml of peptone water (BDH Chemicals Ltd) prepared according to manufacturer’s recommendations. The peptone water was incubated at 36°C for 2 to 5 hours until turbid, and standardized to 0.5 McFarland nephelometer prepared by dissolving 1 gram of barium chloride in 100 ml of sulfuric acid using normal saline. The entire surface of agar was inoculated by swabbing and allowed to dry for 3 to 5 minutes before aseptically placing 4 antibiotic disks per plate. The following commercially prepared antibiotic disks (Oxoid, UK) were used: nitrofurantoin (50 μg), nalidixic acid (30 μg), ciprofloxacin (5 μg), levofloxacin (5 μg), amoxicillin (10 μg), cephalexin (30 μg), amoxicillin-clavulanate (20/10 μg), cotrimoxazole (25/125 μg), gentamicin (10 μg), azithromycin (15 μg) and oxacillin (10 μg). Well set plates were incubated in a dry oven maintained at 35°C (±1°C). Inhibition zone diameters were read at 18 h for all organisms except for Staphylococcus which was read at 24 h. Clear zone diameters were measured to the nearest millimeter using a millimeter ruler under natural light. Internal quality control was ensured by incubating Staphylococcus aureus (ATCC 25923) and Escherichia coli (ATCC 25922) strains under the same conditions.

### Data entry and analysis

Laboratory entries were made in a laboratory book each day. The data was constantly checked for completeness and cleaned during the study. Each measurement of the inhibition zone diameter was interpreted as ‘sensitive’, ‘intermediate’ or ‘resistant’ according to CLSI standard interpretative charts [25]. At the end of collection, data was double-entered into *Epidata*^®^ computer program, validated and analyzed by simple percentages among related variables. For all proportions obtained, confidence intervals set at 95% level of significance were computed using binomial exact distribution.

### Ethical considerations

The study was performed in accordance with the Helsinki Declaration and was approved by the Institutional Review Board of the Faculty of Medicine, Gulu University (reference no. GU/IRB/03/06/11). Permission to conduct the study was granted by the Uganda National Council for Science and Technology. Informed consent was obtained in writing from each participant. The investigators ensured that participants were handled in a professional and culturally sensitive manner and patient confidentiality was observed throughout the study. At the earliest opportunity, clinically important information was forwarded to the attending clinician to ensure optimum patient care.

## Results

### General patient characteristics

Out of 339 samples analyzed over a period of three months (June to August, 2011), only 82 met our inclusion criteria for true UTI. Three samples (3.7%) were obtained from males while seventy nine (96.3%) came from females. Fourteen women (17.7%) were pregnant and one male participant had clinical findings consistent with prostatic hypertrophy. Sixty nine participants (20.4%) reported antibiotic use prior to presenting at the hospital. Most of them (72%) had self-medicated and none had received a full antibiotic course by the time of presentation to the hospital. Table 1 gives a summary of the demographic and clinical characteristics of patients investigated in the study.

**Table 1:** Demographic and clinical characteristics of outpatients with urinary tract infection at Gulu regional hospital (n = 82)

**Parameter**	**Males (n = 3)**	**Females (n = 79)**	**Total (%)**
Mean age in years (range)	46 (37–59)	23 (18–70)	
Pregnancy status among participants	N/A	Not pregnant = 65 Pregnant = 14	79
Presenting symptoms^a^ (%)			
Increased urine frequency	-	85	N/A
Supra pubic pain/discomfort	-	77	N/A
Dysuria	-	68	N/A
Urge incontinence	-	55	N/A
History of recurrent symptoms (in past 6 months)	0	3	3
Immune suppression symptoms	0	0	0
Neurological disease affecting micturition	0	0	0
Renal angle pain/tenderness	0	0	0
Prostatic enlargement	1	N/A	
Recent catheterization	0	0	0
Positive urine sugar	0	0	0
Recent antibiotic use (2 weeks)^b^	0	69	69 (20.4)
Significant leucocyturia (pyuria)^b^	3	115	118 (34.8)
At least 10^5^ CFU/mL^c^	3	64	67 (56.8)

**Key**: *N/A*, Not applicable; *CFU*, Colony forming units. ^a^ calculations only done for female participants as males were too few for meaningful results. ^b ^recorded from all patients having any symptom suggestive of a UTI (n = 339).

^c ^test performed only on patients with significant leucocyturia (n = 118).

### Identity and proportions of uropathogens isolated

As shown in Table 2, Staphylococcal species (n = 38, 46.3%) caused majority of UTI. This was followed by Escherichia coli (n = 32, 39%). Other uropathogens isolated include Enterococcus faecalis (n = 6, 7.3%), Klebsiella pneumoniae (n = 4, 4.9%) and Proteus mirabilis (n = 2, 2.4%). Among the Staphylococcal species isolated, 28 were identified as S. saprophyticus while 9 were identified as S. aureus. There was one case of S. epidermidis isolated from the male patient with prostatic hypertrophy.

**Table 2:** Susceptibility profile of uropathogens isolated from symptomatic outpatients at Gulu regional hospital, northern Uganda (n = 82)

**Uro-isolates**	**Staphylococcus spp. n = 38**			**E. coli n = 32**			**Klebsiella spp. n = 4**			**P. mirabilis n = 2**			**E. faecalis n = 6**		
**Antibiotic disks**	**S**	**I**	**R**	**S**	**I**	**R**	**S**	**I**	**R**	**S**	**I**	**R**	**S**	**I**	**R**
Nitrofurantoin (50 μg)	8	14	16	10	5	17	0	1	3	0	0	2	4	0	2
Nalidixic acid (30 μg)	4	10	24	13	6	13	0	2	2	0	0	2	2	2	2
Cotrimoxazole (25/125 μg)	5	5	28	5	5	22	0	0	4	0	0	2	2	0	4
Amoxicillin (10 μg)	16	6	16	10	3	19	0	1	3	0	2	0	2	0	4
Amoxi/clav (20/10 μg)	26	3	9	10	10	12	0	4	0	0	2	0	3	1	2
Cephalexin (30 μg)	16	13	9	5	8	19	3	1	0	0	0	2	2	0	4
Ciprofloxacin (5 μg)	24	0	14	19	5	8	3	1	0	2	0	0	4	0	2
Levofloxacin (5 μg)	29	2	7	13	9	10	2	2	0	2	0	0	3	1	2
Gentamicin (10 μg)	24	7	7	26	1	5	4	0	0	2	0	0	6	0	0
Azithromycin (15 μg)	6	9	23	10	17	5	4	0	0	1	1	0	5	1	0
Oxacillin (10 μg)*	7	0	2	-	-	-	-	-	-	-	-	-	-	-	-

**Key**: *S*, Sensitive; *I*, Intermediate; *R*, Resistant; * tested on Staphylococcus aureus only (n = 9).

### Antibiotic susceptibility and resistance profiles

Regarding antibiotic susceptibility assays, the zone diameters with all control strains were within published limits. As presented in Table 2, there was widespread resistance among all uropathogens to cotrimoxazole, amoxicillin, nitrofurantoin and nalidixic acid. A summary of the relative proportions of susceptibility and resistance profiles from all uropathogens against each antibiotic is presented in Table 3 along with their confidence intervals set at 95% level of significance. When sensitive and intermediate readings were combined, the most favorable profiles were obtained with gentamicin, amoxicillin-clavulanic acid and levofloxacin for which 85.4%, 72.0% and 67.1% of isolates respectively, were considered susceptible (Table 3). Of the pathogens isolated from male participants, there was one case each, of Escherichia coli, Staphylococcus aureus and Staphylococcus epidermidis. For the entire study, this was the only incidence of Staphylococcus epidermidis. This participant also had symptoms of prostatic hypertrophy. Interestingly as shown in Table 4, this organism had marked sensitivity to most antibiotics tested except nalidixic acid and nitrofurantoin.

**Table 3:** Relative proportions of susceptibility and resistance profiles from all uropathogens isolated from outpatients at Gulu regional hospital, northern Uganda (n = 82)

**Antibiotic (disc potency)**	**Sensitive (95% CI)**	**Intermediate (95% CI)**	**Total **^**a **^**(%)**	**Resistant (95% CI)**
Nitrofurantoin (50 μg)	24 (19.5-40.4)	20 (15.6-35.1)	44 (53.7)	38 (35.3-57.7)
Nalidixic acid (30 μg)	19 (14.7-33.8)	20 (15.6-35.1)	39 (47.6)	43 (41.1-63.6)
Cotrim.^b^ (25/125 μg)	12 (7.8-24.2)	10 (6.0-21.3)	22 (26.8)	60 (56.2-82.4)
Amoxicillin (10 μg)	29 (25.1-46.7)	11 (6.9-22.7)	40 (48.8)	42 (39.9-62.4)
Amox-clav ^c^ (20/10 μg)	39 (36.4-58.9)	20 (15.6-35.1)	59 (72.0)	23 (18.7-39.1)
Cephalexin (30 μg)	26 (21.9-42.9)	22 (17.6-37.8)	48 (58.5)	34 (30.7-52.9)
Ciprofloxacin (5 μg)	36 (33.0-55.3)	6 (2.7-15.2)	42 (51.2)	40 (37.6-60.1)
Levofloxacin (5 μg)	44 (42.3-64.7)	11 (6.9-22.7)	55 (67.1)	27 (22.9-44.2)
Gentamicin (10 μg)	62 (57.4-84.4)	8 (4.3-18.3)	70 (85.4)	12 (7.8-24.2)
Azithromycin (15 μg)	26 (21.9-42.9)	28 (24.0-45.4)	54 (65.9)	28 (24.0-45.4)

**Key: **^a ^sum of sensitive and intermediate readings, ^b ^cotrimoxazole, ^c^ amoxicillin-clavulanic acid.

**Table 4:** Susceptibility profiles of uropathogens isolated from male patients at Gulu regional hospital, northern Uganda (n = 3)

**Uropathogen**	**TS**	**AX**	**CL**	**NI**	**NA**	**CIP**	**LEV**	**CN**	**AZM**	**AMC**	OX*
S. epidermid^a^	S	S	S	R	R	S	S	S	I	S	
S. aureus	R	R	S	I	R	R	R	S	R	S	S
E. coli	R	R	I	S	S	S	R	R	S	R	-

**Key**: *S*, Sensitive; *I*, Intermediate; *R*, Resistant; *TS*, Cotrimoxazole; *AX*, Amoxicillin; *AZM*, Azithromycin; *CL*, Cefalexin; *NI*, Nitrofurantoin; *NA*, Nalidixic acid; *CIP*, Ciprofloxacin; *LEV*, Levofloxacin; *CN*, Gentamicin; *AMC*, Amoxicillin-clavulanic acid; *OX*, Oxacillin, ^a^ Staph. epidermidis was associated with prostatic hypertrophy, * tested on Staphylococcus aureus only.

## Discussion

This study revealed relatively high frequencies of resistance to most antibiotics tested. Resistance to cotrimoxazole, amoxicillin, nitrofurantoin and nalidixic acid were particularly alarming. This is consistent with our clinical observations as well as findings from other recent studies done in Uganda [14,26,27]. Such high levels of resistance are probably due to antibiotic misuse by poorly trained health workers as has been documented in many developing countries [18,28,29]. In addition, weak government regulation allows for the importation and sale of substandard drugs, often from unlicensed outlets. This encourages domiciliary self-medication practices that have become commonplace in Uganda [30,31]. Moreover, it is not uncommon for unqualified health workers to offer clients the option of purchasing small quantities of antibiotics. This leads to inadequate dosing resulting into sub-inhibitory tissue concentrations that facilitate selection of antibiotic-resistant strains. It has been observed that the motives for antibiotic misuse by health workers are similar to those for misuse by lay persons: to cut costs and act expeditiously to treat suspected bacterial infections [28].

The particularly high resistance to cotrimoxazole may be explained by additional factors common in our setting. These include the use of cotrimoxazole as prophylaxis among HIV positive patients [20] and the use of sulfadoxine-pyrimethamine (shares enzyme targets with cotrimoxazole) for routine malaria prophylaxis during pregnancy [20]. In light of these considerations, we strongly suspect that a similar resistance profile occurs in other parts of Uganda. Therefore, UTI treatment with cotrimoxazole is likely to fail, exposing patients to unnecessary distress and an increased risk of complications. Some authors have however questioned the predictive value of in-vitro resistance testing among uropathogens, especially for antibiotics that achieve urinary concentrations much higher than those tested in laboratory assays [6,32]. For instance, it has been reported that urine levels of trimethoprim and sulfamethoxazole are 35 and 3 times, respectively, the levels in serum [33]. In such cases, it is possible that even with apparent in-vitro resistance, successful cure may be realized. In the present context, this issue may require further investigation.

Nearly 50% of uropathogens (40/82) were resistant to ciprofloxacin (Table 3). This is far above the 20% rate recommended for empirical use of antibiotics for community-acquired UTI [34,35]. Ironically, a 32% resistance was found with levofloxacin yet this antibiotic has barely been used in our setting owing to prohibitive costs. A fluoroquinolone cross-resistance phenomenon may explain this finding since the two drugs also share an enzyme target. These findings are of great concern considering that fluoroquinolones are reserved for severe or complicated UTI in Uganda [20]. Our study found a 46% resistance to nitrofurantoin (38/82, Table 3) compared to that of Mwaka et al., where only 2% of isolates were reported resistant among non-pregnant women in Kampala [26]. This apparent disparity might have come from the use of nitrofurantoin disks of different potency (300 μg in Mwaka et al., and 50 μg in ours). In addition, socio-demographic differences between the population in Kampala and Gulu (approximately 400 kilometers apart) might have contributed. Being urban and rural populations respectively, differences in lifestyles, occupation, culture, attitudes and literacy exist. These factors (along with genetics) are known to influence disease and health seeking patterns, and may explain geographical variations in antibiotic resistance even within the same country [16,19].

The most favorable antibiograms were obtained with gentamicin and amoxicillin-clavulanic acid where a total of 85.4% and 72.0% of isolates respectively, were either sensitive or intermediate against all organisms (Table 3). Either of these antibiotics could replace the current choices for empirical treatment of community-acquired UTI in our setting. Gentamicin is quite affordable and is already available within our public hospitals. To mitigate toxicity concerns, our physicians have adopted the newer once daily dosing of gentamicin known to be safer yet as effective as multiple dosing schedules [36]. An important drawback however is the fact that gentamicin has to be administered exclusively by injection, and a move towards its adoption is likely to be material and labor intensive. We fear that a resource-constrained healthcare system like ours will be particularly sensitive to additional strain. On the other hand, the high cost of oral amoxicillin-clavulanate means that it may not be available to all those who need it within the public sector.

At 46.3%, Staphylococcal species formed the majority of uropathogens isolated in our study. This was followed by Escherichia coli at 39%. This pattern is slightly different from that seen in most literature, where Escherichia coli commonly predominates [6-13]. Our findings are however similar to those reported by Kyabaggu et al. [14], where Staphylococcal species (32%) followed by Klebsiella (21%) were the commonest uropathogens isolated among patients at a metropolitan hospital in Kampala. Two other studies done at Mulago national referral hospital in Kampala ranked Staphylococcal species second to Escherichia coli among leading causes of asymptomatic bacteriuria among pregnant and non-pregnant women [26,27]. In our study, most Staphylococcal infections were due to S. saprophyticus (28/38). This organism has generally been found to colonize the urinary tract of sexually active women [37,38]. Our findings may point in this direction and are implicitly supported by the young study population and the fact that Uganda has one of the highest fertility rates in the world [39]. With a mean age 23 years, female participants in our study were much younger than female participants in a similar European study (mean age 51.6) published recently [40]. Looking at these two populations, differences in sexual patterns that may influence UTI etiology are likely to exist.

This study presents outpatient-based antibiograms of uropathogens isolated from consecutively selected participants presenting to Gulu regional hospital. This represents a fairer picture of antibiotic susceptibility profiles within this community than would be expected from inpatient-based (or laboratory-based) antibiograms. The latter are more likely to reflect a selection bias towards complicated UTI and therefore, to over-estimate resistance prevalence in a particular community [32]. In this study, the proportion of samples that turned out as true UTI was rather small (82/339) compared to the participant number that were recruited with symptoms. The poor correlation between symptoms and positive diagnosis may be explained by the rampant tendency among patients to self-medicate and only visit hospitals when symptoms persist. In such cases, quantitative urine culture is likely to be less sensitive, leaving investigators with a diagnostic dilemma. Consequently, our decision to adhere to the 10^5^ CFU/mL cut-off value might have excluded a significant proportion of UTI cases. In addition, the decision to consider as contaminants, all suspected cases where two or more organisms were isolated might also have contributed to the low numbers observed. Nevertheless, we were able to isolate most species commonly associated with community-acquired UTI in reasonable proportions. As shown in Table 1, some urine samples with leucocyturia did not yield significant bacterial growth on quantitative culture. Further investigations revealed presence of yeast cells in these samples, suggesting a fungal infection. Some urine samples did not yield any significant findings using our criteria despite symptoms. We think these symptoms were due to obstetric or other gynecological diseases that the study did not set out to investigate.

## Conclusion

Staphylococcal species and E. coli are major causes of community-acquired UTI in our setting. There is unacceptably high resistance to cotrimoxazole and amoxicillin among uropathogens isolated in our setting. Continued use of these drugs is likely to be associated with a high risk of treatment failure. The recommendations of the Uganda Clinical Guidelines, 2010 are not in tandem with the situation obtaining on the ground in Gulu district. There is need to formulate an appropriate hospital antibiotic policy for the treatment of UTI in our setting. In the meantime, clinicians should take these findings into account whenever prescribing empirically for UTI. We recommend that either gentamicin or amoxicillin-clavulanate be used for empirical treatment of UTI in our setting.

## Competing interests

The authors declare that they have no competing of interests.

## Authors’ contribution

COO and DAA conceived the design of the study with contribution from KL and PO. COO and DAA were involved in training research assistants, data collection and clinical care. COO, DAA and KL performed the laboratory investigations. COO drafted the manuscript and DAA, KL and PO contributed to its revision. All authors read and approved the final manuscript.

## Pre-publication history

The pre-publication history for this paper can be accessed here:

http://www.biomedcentral.com/1471-2334/13/193/prepub
